# Growth and crystallographic feature-dependent characterization of spinel zinc ferrite thin films by RF sputtering

**DOI:** 10.1186/1556-276X-8-537

**Published:** 2013-12-19

**Authors:** Yuan-Chang Liang, Hao-Yuan Hsia

**Affiliations:** 1 Institute of Materials Engineering, National Taiwan Ocean University, Keelung 20224, Taiwan

**Keywords:** Spinel, Oxide, Crystallographic feature, Sputtering, Surface morphology, Magnetic property

## Abstract

ZnFe_2_O_4_ (ZFO) thin films exhibiting varying crystallographic features ((222)-epitaxially, (400)-epitaxially, and randomly oriented films) were grown on various substrates by radio-frequency magnetron sputtering. The type of substrate used profoundly affected the surface topography of the resulting ZFO films. The surface of the ZFO (222) epilayer was dense and exhibited small rectangular surface grains; however, the ZFO (400) epilayer exhibited small grooves. The surface of the randomly oriented ZFO thin film exhibited distinct three-dimensional island-like grains that demonstrated considerable surface roughness. Magnetization-temperature curves revealed that the ZFO thin films exhibited a spin-glass transition temperature of approximately 40 K. The crystallographic orientation of the ZFO thin films strongly affected magnetic anisotropy. The ZFO (222) epitaxy exhibited the strongest magnetic anisotropy, whereas the randomly oriented ZFO thin film exhibited no clear magnetic anisotropy.

## Background

Recently, spinel-structured ferrite oxides have been intensively investigated because of their versatile physical and chemical properties as well as technological applications in magnetic sensors, biosensors, and photocatalysts [1,2]. ZnFe_2_O_4_ (ZFO) is one of the major ferrite oxides with a spinel structure, and it has remarkable magnetic and electromagnetic properties regarding its state of chemical order and cation site occupancy in lattices [3]. Moreover, it is also a semiconductor, processes light response, has photochemical characteristics, and can be used as a material for supercapacitors [4,5].

ZFO in various forms, such as powders, films, and various nanostructures, prepared using different methodologies have been reported [6-8]. Many ZFO nanostructures can be used as versatile building blocks for fabricating functional nanodevices; however, integrating the reported methodologies for preparing nanostructured ZFO into Si-based semiconductor device processes remains a challenge. ZFO in thin-film form is promising and is compatible in the fabrication of devices with Si semiconductors. Yamamoto et al. prepared ZFO thin films on a single-crystal sapphire substrate by using pulsed laser deposition and examined the effect of the deposition rate on its magnetic properties [9]. ZFO thin films with a microlevel scale were grown on glass substrates by radio-frequency (RF) sputtering at room temperature, and the magnetic properties of the films were investigated [10]. Ogale et al. used a pulsed laser evaporation method to synthesize ZnO and Zn_*x*_Fe_3−*x*_O_4_ mixed-phase thin films on sapphire substrates using ZnFe_2_O_4_ pellets; however, this is not an efficient method for obtaining single-phase spinel ZFO thin films [11]. Polycrystalline ZFO films were also prepared by spin-spray deposition; however, controlling the film thickness to be less than several hundred nanometers is challenging [12]. Although several groups have proposed the fabrication of ZFO films using versatile methodologies, the sputtering technique is promising for preparing oxide thin films with excellent crystalline quality and controllable film thickness for device applications because it is a technique that enables large-area deposition and easy process control [13,14]. It is well known that crystallographic features affect the properties of versatile oxide films [13,15]. However, the crystallographic feature-dependent properties of sputtering-deposited spinel ZFO thin films are still inadequate. This might obstruct applications of such films in devices. In this study, ZFO thin films were grown on various single-crystal substrates by RF sputtering to fabricate ZFO thin films with varying crystallographic features. The correlation between the crystallographic features and the characterization of the ZFO thin films was investigated.

## Methods

ZnFe_2_O_4_ (ZFO) thin films were grown on yttria-stabilized zirconia (YSZ) (111), SrTiO_3_ (STO) (100), and Si(100) substrates, using RF magnetron sputtering. The yttria content in YSZ substrates was 15%. The sputtering ceramic target adopted in the experiment was prepared by mixing the precursor oxide powders of ZnO and Fe_2_O_3_ to obtain a proportion of Fe/Zn = 2, pressing the powders into a pellet, and sintering the pellet at a high temperature to achieve a high density. The thickness of the ZFO thin films was fixed at approximately 125 nm, and the growth temperature was maintained at 650°C. The gas pressure of deposition was fixed at 30 mTorr, using an Ar/O_2_ ratio of 2:1 for the films. The atomic percentages of the as-deposited films were calculated based on the X-ray photoelectron spectroscopy (XPS) spectra of the Zn2p, Fe2p, and O1s regions. The chemical binding states of the constituent elements of the ZFO thin films were also investigated.

The crystal structures of the samples were investigated using X-ray diffraction (XRD), applying Cu Kα radiation. The surface morphology of the ZFO films was determined using scanning electron microscopy (SEM) and atomic force microscopy (AFM) at an area of 1 μm^2^. The detailed microstructures of the as-synthesized samples were characterized using high-resolution transmittance electron microscopy (HRTEM). The composition analysis was performed using an energy-dispersive X-ray spectrometer (EDS) attached to the TEM. Thin slices for cross-sectional TEM analysis were prepared using a dual-beam focused-ion-beam (FIB) instrument. The areas selected for cutting with an ion beam were protected by an amorphous carbon overlayer. Adjust the beam currents to mill initial trenches, thin the central membrane, and polish for electron transparency of membrane. Finally, FIB milling was used to capture a free membrane from trenches for a TEM analysis. The room temperature-dependent photoluminescence (PL) spectra were captured using the 325-nm line of a He-Cd laser. A superconducting quantum-interference device magnetometer was used to measure the magnetic properties of the samples.

## Results and discussion

**Figure 1:**
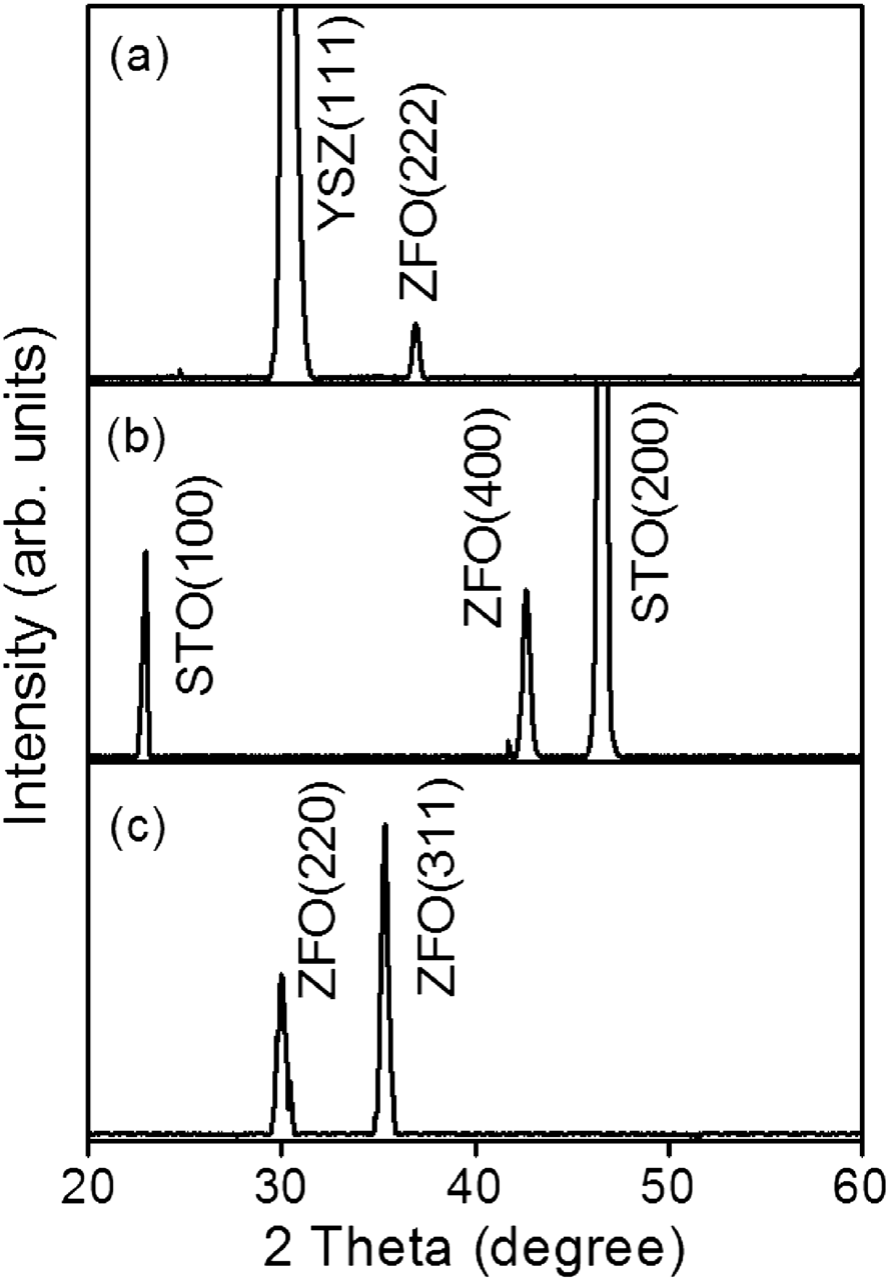
**XRD patterns of the ZFO thin films on various substrates: (a) YSZ (111), (b) SrTiO**_**3 **_**(100), and (c) Si (100).**

Figure 1 displays the X-ray diffraction (XRD) patterns of the ZFO thin films grown on various substrates. The XRD patterns show several sharp and intense Bragg reflections originating from the ZFO structure (according to JCPDS No. 89–1012), confirming that the ZFO thin films exhibited excellent crystalline quality. The absence of ZnO and Fe_*x*_O_*y*_ phases in the XRD patterns indicated that an exceptional ZFO compound was formed. The ZFO films grown on the YSZ and STO substrates exhibited highly (222) and (400) crystallographic orientations, respectively. By contrast, the film grown on the Si substrate was randomly oriented. Most of the grains on the ZFO thin film grown on the Si substrate were (311)-oriented and some were (220)-oriented. The lattice constants of the ZFO thin films were derived from the observed Bragg reflections and were independent of the substrate types used in this study. The lattice constants of the ZFO thin films were approximately 0.843 nm, and this value was similar to that of its bulk counterpart (approximately 0.844 nm) [16], indicating that the highly oriented ZFO thin films were not affected by lattice distortion of the substrates (caused by a lattice mismatch between film and substrate). This might be attributed to the film thickness (approximately 125 nm), which markedly exceeded the critical value for misfit strain relaxation [17,18].

**Figure 2:**
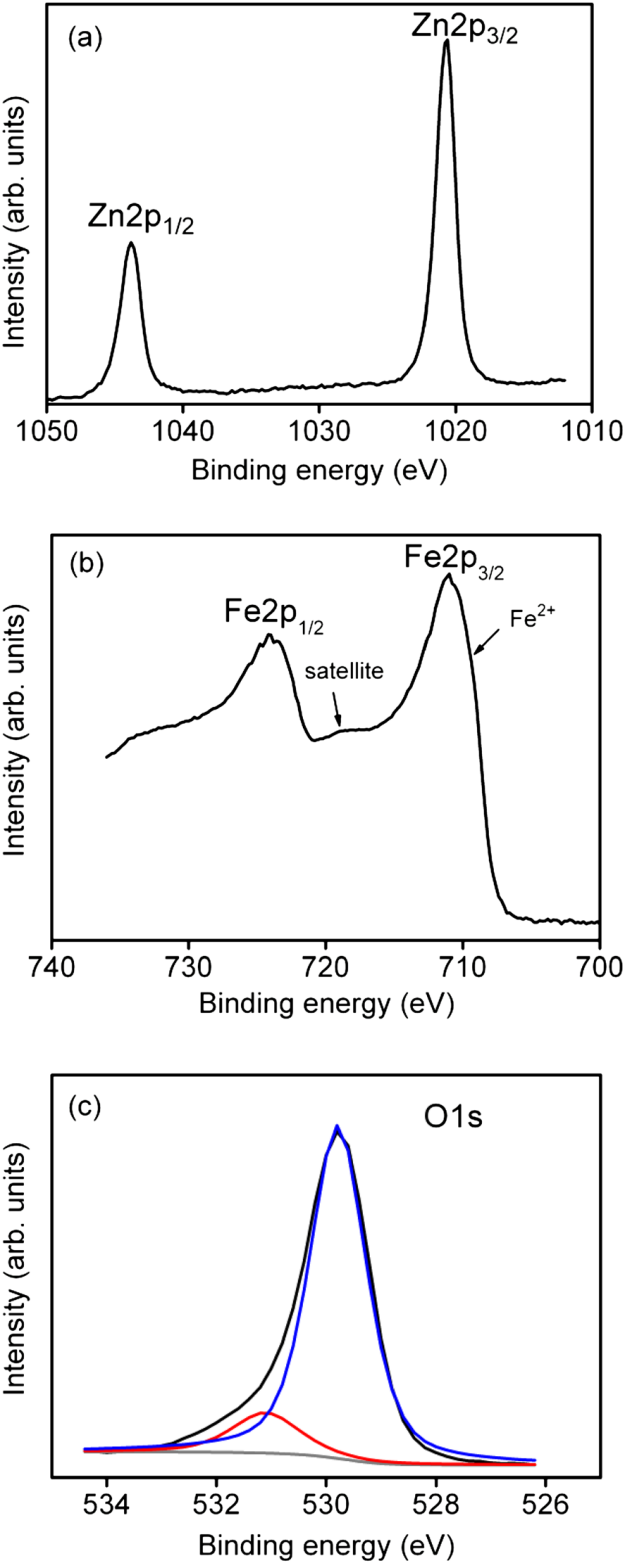
**Narrow-scan XPS spectra of the constituent elements in the ZFO thin film. (a)** Zn 2p core**-**level, **(b)** Fe 2p core**-**level, and **(c)** O1s core**-**level.

The atomic percentage of the Fe/Zn and binding states of the Zn and Fe constituent elements for the as-deposited ZFO thin film was evaluated based on the narrow-scan XPS spectra of Zn and Fe. The Fe/Zn atomic ratio was approximately 2.04, and this ratio is similar to the Fe/Zn stoichiometric composition of the ZFO. Figure 2a shows a Zn2p narrow-scan XPS spectrum. The binding energies of Zn2p_3/2_ and Zn2p_1/2_ were 1,020.7 and 1,043.7 eV, respectively. These binding energies are close to the reported values of the binding state of Zn^2+^[19]. The core-level spectrum of Fe had a 2p_3/2_ binding energy of approximately 711.1 eV (Figure 2b). Moreover, a clear broad shake-up satellite of binding energy at approximately 719.1 eV was observed. The energy difference between the 2p_3/2_ and 2p_1/2_ was approximately 13 eV in this study. These features were mainly associated with the Fe^3+^ binding state in the ZFO [20]. A shoulder at approximately 709.5 eV was observed in the Fe-XPS spectrum, which might be associated with iron atoms in the ZFO lattices that were bonded in Fe^2+^ status [21]. A symmetric O1s spectrum was observed for the as-deposited ZFO thin film (Figure 2c). The Gaussian-resolved results showed that the spectrum consisted of two peak components. The first was centered at approximately 529.7 eV and was attributed to the oxygen in the ZFO crystal. The second was centered at approximately 531.1 eV, representing the oxygen ions in the oxygen-deficient regions. The formation of oxygen vacancies in the sputtered ZFO thin films was attributed to the oxygen-deficient environment during thin-film preparation [22]. The nonstoichiometric oxygen content in the ZFO thin film supported the observation of the Fe-core-level spectrum that Fe^2+^ and Fe^3+^ coexisted in the ZFO.

**Figure 3:**
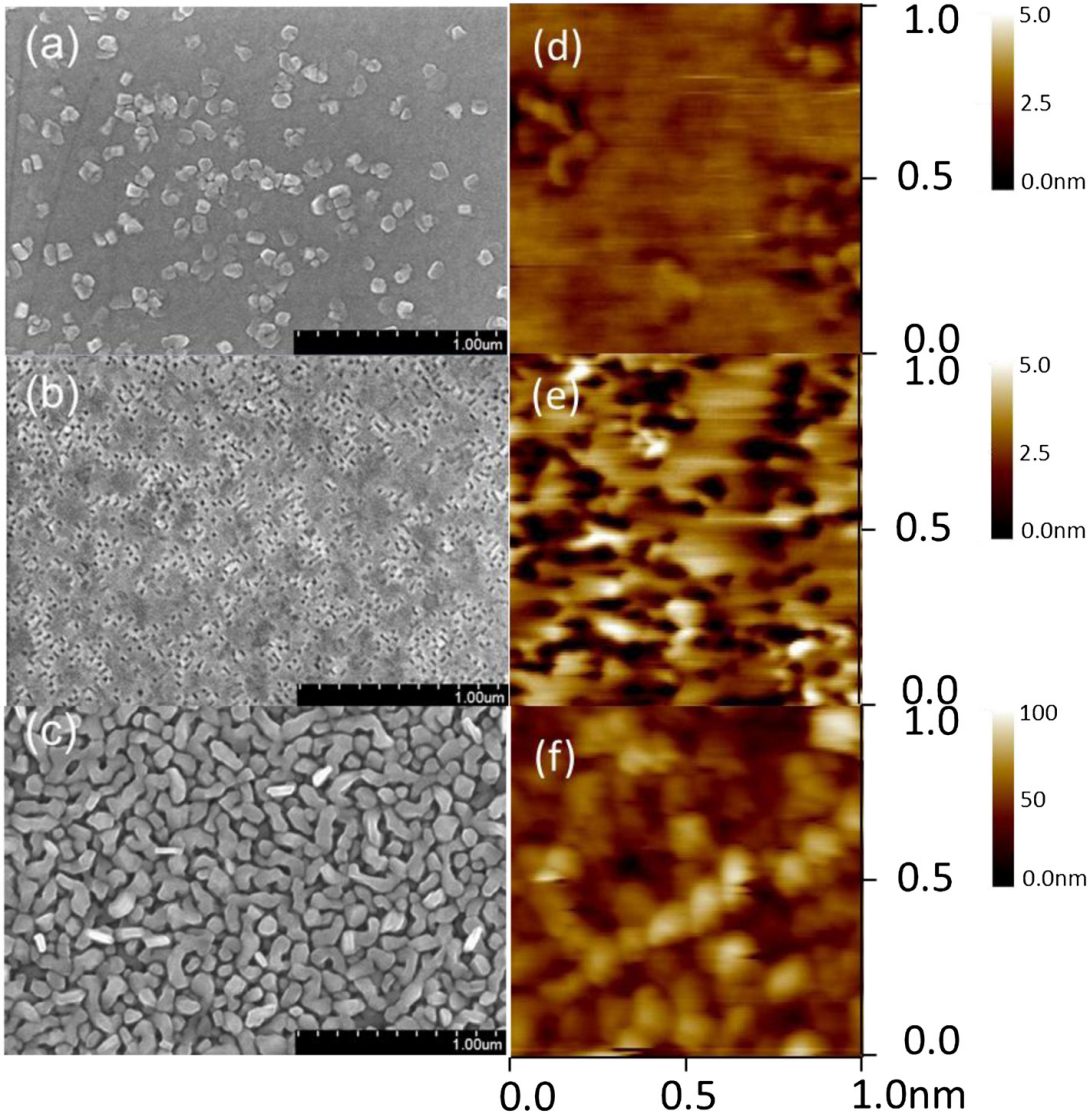
**SEM images of the ZFO thin films grown on various substrates. (a)** YSZ (111), **(b)** SrTiO_3_ (100), and **(c)** Si (100) and AFM images: **(d)** YSZ (111), **(e)** SrTiO_3_ (100), and **(f)** Si (100).

Figure 3 shows the SEM images of the ZFO thin films grown on the various substrates. The morphologies of the ZFO thin films differed depending on the substrate on which they were grown. The surface of the ZFO grown on the YSZ substrate was dense and comprised tiny grains (Figure 3a). Most of the grains were in a rectangular morphology with a size of approximately 100 to 130 nm. The surface of the ZFO film grown on the STO substrate consisted of numerous tiny grooves (Figure 3b). These grooves were approximately 20 to 30 nm. Clear three-dimensional (3D) bar-like grains homogeneously covered the surface of the film grown on the Si substrate (Figure 3c). The size range of these bar-like grains was 150 to 200 nm; these grains were large in comparison with those of the other samples. The detailed surface microstructures of the ZFO thin films were further analyzed by using an atomic force microscope (AFM). A considerable portion of the surface of the ZFO thin film grown on the YSZ substrate was observed to be flat and had a root-mean-square (RMS) surface roughness of 0.49 nm (Figure 3d). The many dark spots distributed over the AFM surface image indicated that numerous tiny sunken regions were present on the ZFO surface (Figure 3e). This surface feature contributed to an RMS surface roughness of 1.19 nm on the STO. Figure 3f shows spiral-shaped surface grains covering the surface of the ZFO thin film grown on the Si substrate. The distinct 3D granular structure of this ZFO surface caused the surface to be relatively rough. The RMS surface roughness was 15.21 nm.

**Figure 4:**
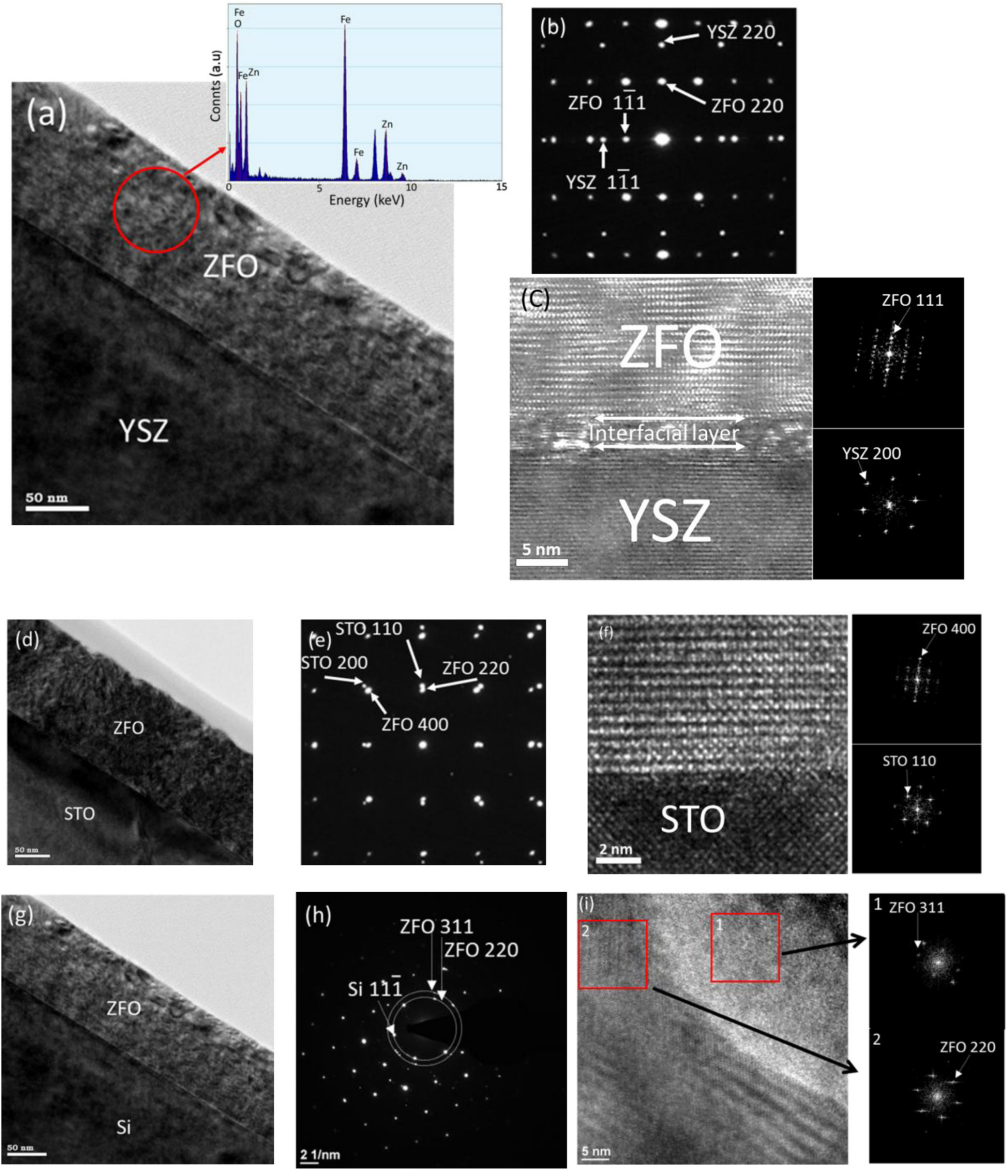
**TEM analysis results of the ZFO film on the YSZ, STO, and Si. (a)** Low-magnification TEM image of the ZFO film on the YSZ. The EDS spectra taken from the film were also displayed. **(b)** The selected area electron diffraction pattern from the ZFO film and YSZ. **(c)** HRTEM images and corresponding FFT patterns taken from the various regions of the sample. **(d)** Low-magnification TEM image of the ZFO film on the STO. **(e)** The selected area electron diffraction pattern from the ZFO film and STO was also presented. **(f)** HRTEM image taken from the ZFO film-STO interfacial region. **(g)** Low-magnification TEM image of the ZFO film on the Si. **(h)** The selected area electron diffraction pattern from the ZFO film and Si. **(i)** HRTEM images and corresponding FFT patterns taken from the ZFO film grown on the Si.

The low-magnification cross-sectional transmission electron microscopy (TEM) image (Figure 4a) of the ZFO thin film grown on the YSZ substrate revealed a dense and flat film with no macroscopic imperfection; the total thickness of the ZnO layer was approximately 125 nm. The EDS analysis in Figure 4a confirmed the presence of Zn, Fe, and O in the film, and the atomic ratio of Fe/Zn (2.02) was close to the stoichiometric ratio of the ZFO. The clear and ordered spots in the electron diffraction pattern (DP) taken from the film-substrate region (Figure 4b) exhibited that the growth of the ZFO film on the YSZ substrate was <111 > _ZFO_//<111 > _YSZ_ and <110 > _ZFO_//<110 > _YSZ_. Figure 4c presents the cross-sectional high-resolution (HR) TEM image of the ZFO film grown on the YSZ substrate; the corresponding fast Fourier transform (FFT) patterns captured from the ZFO film, film-substrate interface, and YSZ are also shown in the insets. The interface between the ZFO and the YSZ contained a thin transition layer. Above this layer, an ordered atomic arrangement was observed, revealing epitaxial growth of the ZFO on the YSZ substrate. Figure 4d shows the low-magnification cross-sectional TEM image of the ZFO film grown on the STO substrate. The film was dense; however, several tiny grooves were observed on the film surface, and this resulted in a more rugged surface compared with that of the film grown on the YSZ substrate. The DP pattern taken from the film-substrate region is shown in the inset of Figure 4d, which revealed that the growth of the ZFO film on the STO substrate was <100 > _ZFO_//<100 > _STO_ and <110 > _ZFO_//<110 > _STO_. The HR image (Figure 4e) showed that the ZFO had clear and ordered lattice fringes, indicating that the film was of high crystalline quality and that the interface between the ZFO and STO was atomically sharp; no intermediate phase was observed at the interface. By contrast, for the ZFO grown on the Si substrate, the low-magnification TEM image (Figure 4f) reveals that the ZFO film consisted of a clear column-like structure. The surface was rough. The DP pattern comprised ordered spots from the Si and many tiny randomly distributed spots and rings from the ZFO film. The ZFO film had a polycrystalline structure. The HR image and FFT patterns in Figure 4g show that the ZFO grains had different crystallographic orientations, and clear boundaries were present among the grains. According to the results of TEM analyses, the ZFO thin film grown on the Si substrate was more structurally defective than were the ZFO (222) and ZFO (400) epitaxial films.

**Figure 5:**
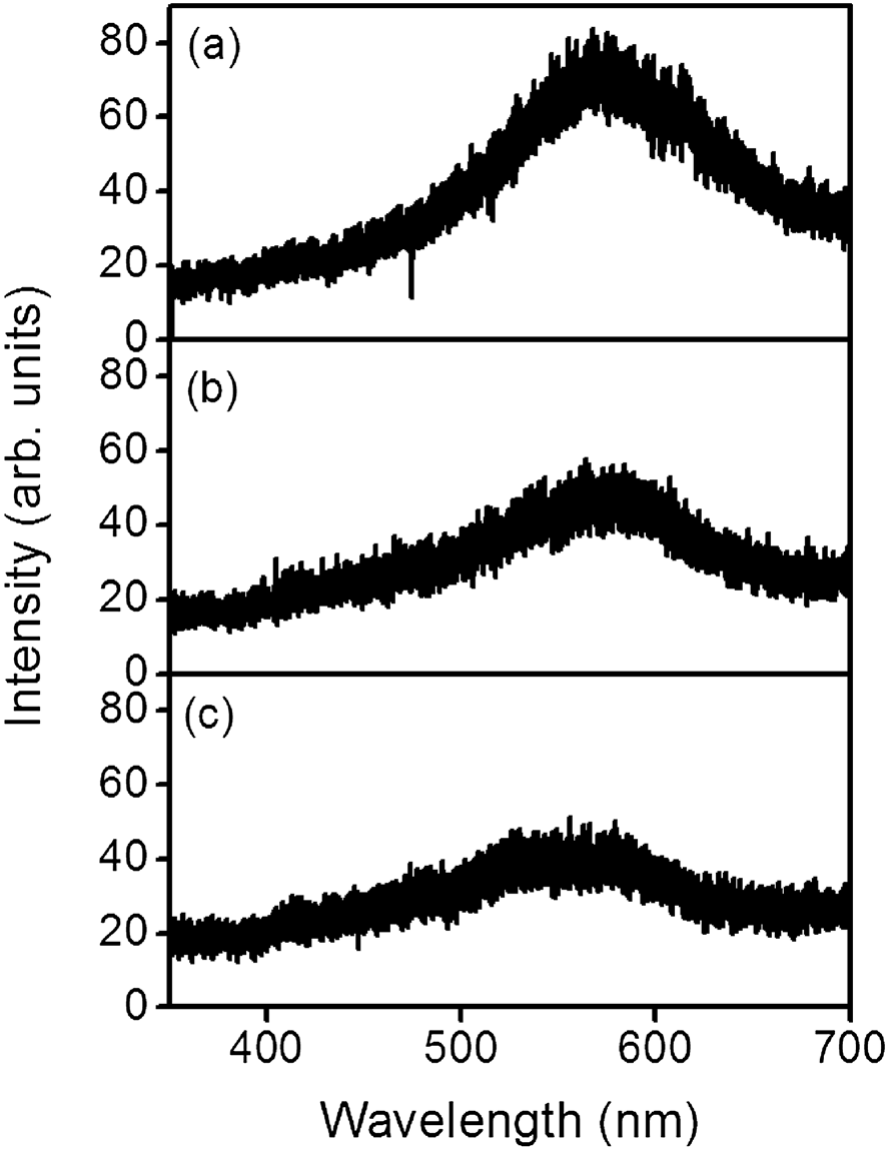
**PL spectra of the ZFO thin films grown on various substrates: (a) YSZ (111), (b) SrTiO**_**3 **_**(100), and (c) Si (100).**

Figure 5 shows the room-temperature photoluminescence spectra of the ZFO thin films grown on the various substrates. A broad peak in the visible emission range and a maximum of approximately 560 to 580 nm were observed for the ZFO thin films. A blue emission band at approximately 468 nm was observed in the Zn-Fe-O compound that had interstitial zinc defects [23]. In the XPS analysis, a symmetrical Zn2p spectrum revealed that there were no excess Zn interstitials in the ZFO lattices, and hence, no such blue emission band was observed in this study. A similar broad visible band, which was attributed to deep-level emissions caused by surface-oxygen-related defects, has been widely reported in ZnO oxides [24]. Insufficient oxygen in the sputtering process generates oxygen vacancies in the ZFO oxide during crystal growth, and this might have caused surface defects in the film, further inducing a yellow emission band.

**Figure 6:**
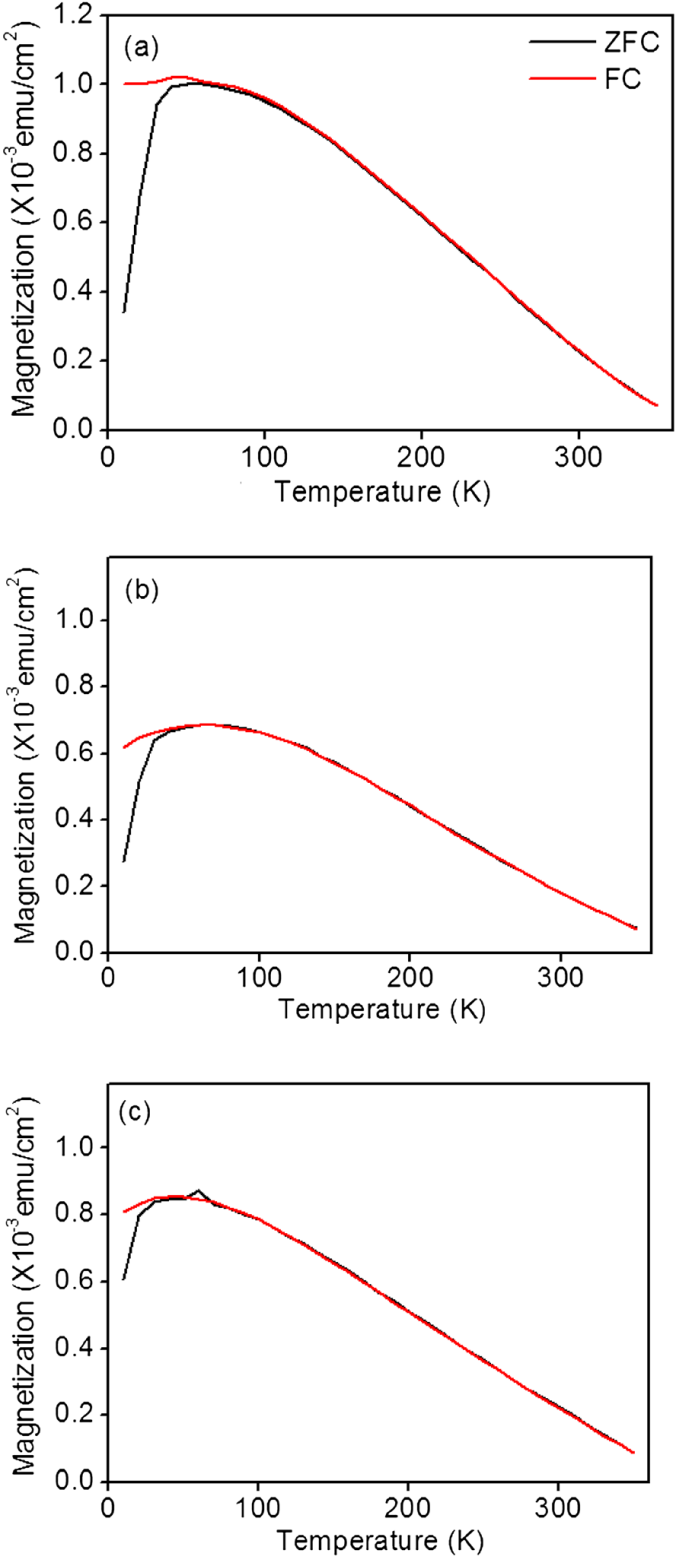
***M*-*T *** **curves of the ZFO thin films grown on various substrates: (a) YSZ (111), (b) SrTiO**_**3 **_**(100), and (c) Si (100).**

Figure 6a,b,c shows the relationship between temperature (*T*) and magnetization (*M*) (zero-field-cooled (ZFC) and field-cooled (FC)) for the ZFO thin films. The *M*-*T* curves were similar among the samples. The observed increase in the *M* of all samples as the temperature decreased was caused by stronger *A*-*B* interaction at lower temperatures in Zn-Fe-O lattices [25]. A non-zero *M* value was observed up to the maximum measurement temperature (350 K) in this study. The ZFC and FC curves showed great differences in the samples below 40 K. The ZFC curves showed a broad peak with a clear summit region. This proved that the films were in a cluster glass state [26]. The spin-glass transition temperature was observed to be nearly 40 K in this study, which is in agreement with results reported in the literature [27]. The bulk ZFO had a spin-glass transition temperature (*T*_g_) of 20 to 30 K. The ZFO thin film had a slightly higher *T*_g_ value than did the bulk ZFO. This was attributed to the disordered cation distribution of Zn^2+^ and Fe^3+^ ions in the spinel structure [10]. Moreover, the random configuration of zinc and iron ions of the spinel structure was associated with oxygen vacancies in the lattices [9]. The XPS analysis results showed that the sputtering-deposited ZFO thin films herein had some degree of oxygen vacancy, which might have contributed to the observed M-T results.

**Figure 7:**
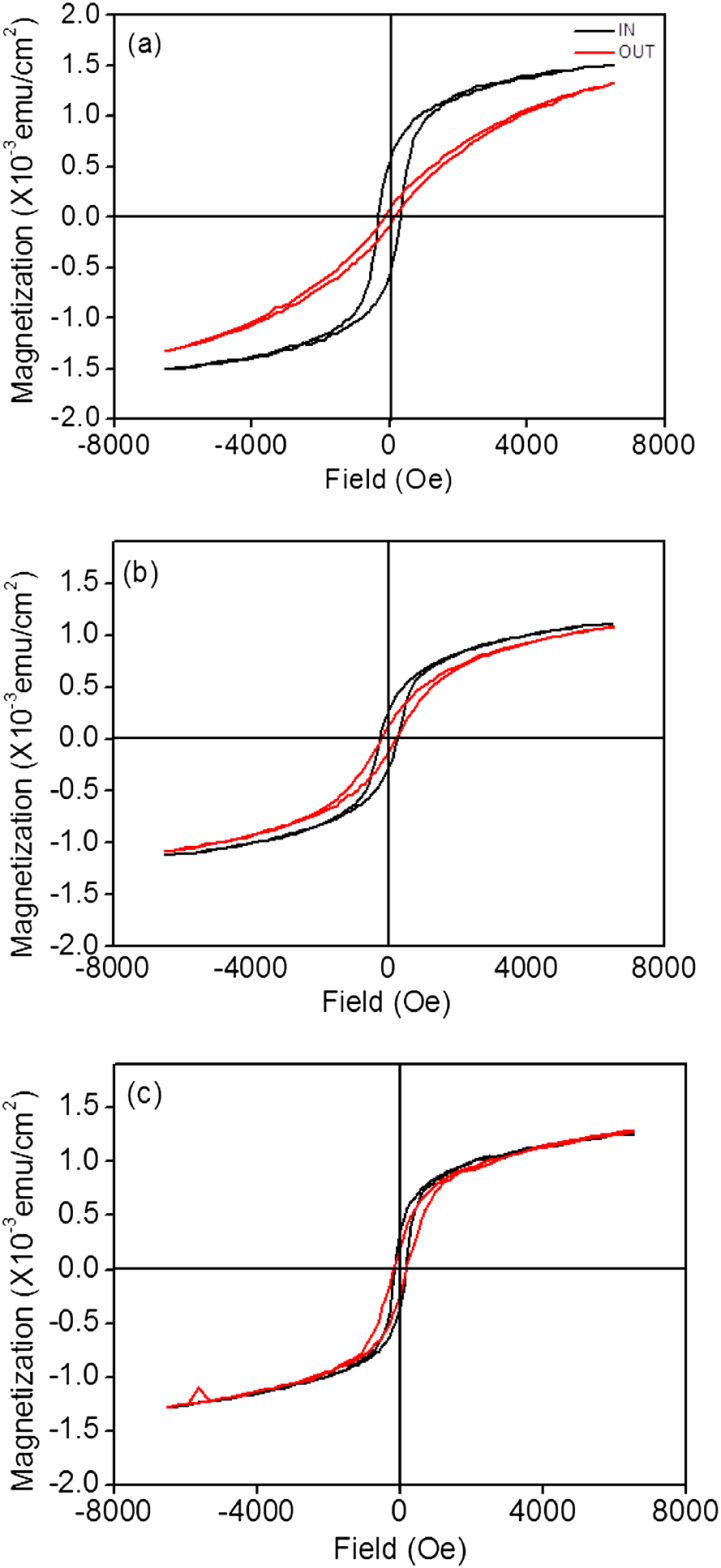
***M*-*H *** **curves of the ZFO thin films grown on various substrates: (a) YSZ (111), (b) SrTiO**_**3 **_**(100), and (c) Si (100).**

Figure 7a,b,c displays the magnetization loops of the ZFO thin films grown on various substrates. The magnetic hysteresis loops were recorded at 30 K with the applied field *H* parallel (*H*_*p*_) and perpendicular (*H*_*v*_) to the film surface. At a measurement temperature of 30 K, the remanence was evident for all samples. Up to 6,500 Oe, the magnetization was far from being saturated. The *M*-*H* behavior clearly showed ferromagnetic coupling because of the A-O-B superexchange interaction. Some Fe^3+^ ions occupied the tetrahedral A-sites and activated the A-B superexchange interaction in the mixed spinel type [28]. When the field was applied parallel to the film surface, the magnetic hysteresis of the ZFO thin film grown on the YSZ substrate was more square than that of the films grown on the STO and Si substrates. The remnant magnetization was 5.5 × 10^−4^ emu/cm^2^, and the coercive field was 311 Oe. Moreover, when the field was applied perpendicular to the film surface, the hysteresis loop of the ZFO (222) epitaxy was the least square among those of all of the samples. The remnant magnetization was 8.2 × 10^−5^ emu/cm^2^, and the coercive field was approximately 140 Oe. The difference in the coercive field values when the field was parallel and perpendicular to the film surface was immense for the ZFO (222) epitaxy, whereas that for the randomly oriented ZFO thin film was small (randomly oriented ZFO thin film: *H*_c*p*_ = 161 Oe and *H*_c*v*_ = 171 Oe). The magnetic hysteresis loops in parallel and perpendicular directions were separating, indicating the presence of magnetic anisotropy for the ZFO thin films on the various substrates. The ZFO (222) epitaxy exhibited the strongest magnetic anisotropy. For the spinel ferrite, the easy axis of magnetization was <100>, and the difficult axis was <111 > [29]. When the field was applied perpendicular to the surface of the ZFO (222) epitaxial film, the field was parallel to the difficult magnetization axis [222] of the ZFO. This caused a less-square magnetic hysteresis loop of the ZFO (222) epitaxial film compared with that when the field was applied parallel to the film surface. A similar magnetic hysteresis loop was observed for the ZFO thin film grown on the Si substrate when the field was applied parallel and perpendicular to the film surface. This was attributed to the random orientation of the magnetic grains in the thin film [30]. This was supported by the structural analyses that the ZFO thin film grown on the Si substrate had a random crystallographic feature.

## Conclusions

ZFO spinel thin films exhibiting epitaxially and randomly oriented crystallographic features were grown on various substrates by RF magnetron sputtering at 650°C. The XRD and TEM results indicated that growing the ZFO thin films on the YSZ (111) and STO (100) substrates promoted the formation of (222) and (400) epitaxial films, respectively. The film grown on the Si substrate exhibited a polycrystalline structure. The surface morphology of the ZFO thin film substantially depended on its crystallographic features. The SEM and AFM images demonstrated that the surface of the ZFO (222) epitaxial film was flat and smooth; however, the surface of the randomly oriented film was rough and exhibited 3D grains. The visible emission bands of the ZFO thin films were attributed to growth-induced oxygen vacancies. The ZFO thin films demonstrated a spin-glass transition temperature of approximately 40 K. The ZFO (222) epitaxial film exhibited the most marked magnetic anisotropy among the samples.

## Competing interests

The authors declare that they have no competing interests.

## Authors’ contributions

YCL designed the project of experiments, analyzed and interpreted the data, and drafted the manuscript. HYH carried out the thin-film preparation and materials analyses. Both authors read and approved the final manuscript.
