# Correction: Growth and crystallographic feature-dependent characterization of spinel zinc ferrite thin films by RF sputtering

**DOI:** 10.1186/1556-276X-9-313

**Published:** 2014-06-25

**Authors:** Yuan-Chang Liang, Hao-Yuan Hsia

**Affiliations:** 1 Institute of Materials Engineering, National Taiwan Ocean University, Keelung 20224, Taiwan

## Correction

Figure 4g in the original version of this article [1] was misused in the typesetting process. The figure 4g is the same as figure 4a. The corrected image for figure 4g is shown here (Figure 1).

**Figure 1:**
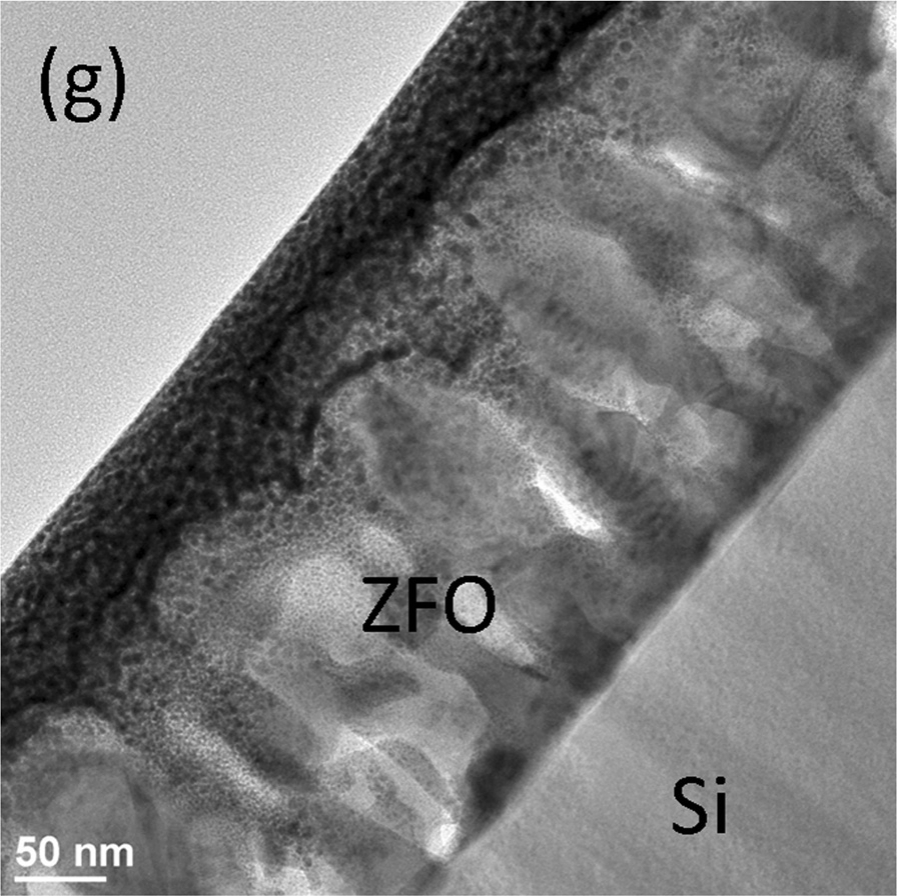
Low-magnification TEM image of the ZFO film on the Si.
